# Percutaneous Surgery for Tailor's Bunion: A Comparative Study

**DOI:** 10.1055/s-0043-1776292

**Published:** 2023-12-05

**Authors:** Luiz Carlos Ribeiro Lara, Lara Furtado Lancia, Diego Vitor Braga Santos, Matheus Maciel Dornelles de Carvalho, Frederico Pinheiro de Lima, João Lucas Gonçalves Arruda

**Affiliations:** 1 Serviço de Ortopedia e Traumatologia, Hospital Municipal Universitário de Taubaté, Taubaté, SP, Brasil

**Keywords:** bunion, metatarsal bones, bunion, tailor's/diagnostic imaging, bunion, tailor's/surgery, treatment outcome

## Abstract

**Objective** To analyze and compare the clinical and radiographic outcomes of bunionette correction using two percutaneous surgical techniques: the Sponsel technique and the medial wedge osteotomy of the distal metaphysis. The results were evaluated individually and comparatively using the American Orthopaedic Foot and Ankle Society's Lesser Metatarsophalangeal-Interphalangeal Scale (AOFAS), Visual Analog Scale (VAS) for pain assessment, and radiographic measurements of the intermetatarsal angle IV-V (AIM4–5) and metatarsophalangeal angle of the fifth ray (AMF-5).

**Methods** This was a retrospective study conducted from May 2011 to February 2022. A total of 32 feet were operated on, with 12 feet undergoing the Sponsel technique and 20 feet undergoing the medial wedge osteotomy of the distal metaphysis of the fifth metatarsal.

**Results** Both surgical techniques showed significant improvement in the correction of AIM4–5 and AMF-5 angles ( *p* < 0.001). However, there was no statistical significance when comparing the two techniques. In terms of AOFAS and VAS scores, both techniques yielded satisfactory results. Nevertheless, the medial wedge osteotomy demonstrated significantly better outcomes compared with the Sponsel technique ( *p* < 0.001).

**Conclusions** Both percutaneous techniques employed for bunionette correction resulted in significant improvement in radiographic angles and evaluated scores, with a low complication rate, making them viable options for treating this condition. When compared, the medial wedge osteotomy appeared to yield better clinical outcomes.

## Introduction

Tailor's bunion is characterized by a deformity in the region of the head of the fifth metatarsal with painful lateral, dorsolateral or plantar prominence. Its etiology is multifactorial, 1 being associated with both anatomical and structural factors. 2 3 4

The initial treatment involves implementing conservative measures, 5 while surgical treatment is indicated when conservative treatment fails. Several surgical techniques have been described for the treatment of this deformity, which basically consist of exostectomies, bone resections and metatarsal osteotomies (distal, diaphyseal and proximal). These techniques are indicated after a clinical and radiographic evaluation, based on the classification of the type of deformity. 1 2 4 6 7

Initially described by Isham 8 and De Prado et al., 9 percutaneous surgery has been gaining space and popularity in recent years, proving to be a useful procedure for treating tailor's bunion in a minimally invasive way. 7

The objective of the present study is to compare the clinical and radiographic aspects of patients undergoing surgical treatment for tailor's bunion using two different percutaneous techniques: Sponsel's oblique osteotomy 1 and medial wedge osteotomy of the distal metaphysis of the 5th metatarsal bone (MWO). 5

## Methods

During the period between May 2011 and February 2022, 29 patients with Tailor's Bunion were operated on, 3 of which were bilateral, thus totaling 32 feet. The procedures were performed at a Municipal University Hospital of Taubaté and in a private clinic owned by one of the authors.

This study included symptomatic cases refractory to conservative treatment, with a minimum follow-up time of 6 months postoperatively. Furthermore, patients with the following characteristics were excluded: history of previous surgery on the 5th ray, those with vascular and/or rheumatoid diseases and those with calluses on the 5th metatarsal bone that did not generate radiographic angular changes, these being submitted only to exostectomy.

In this context, among the 32 feet with tailor's bunion evaluated in the study, other associated pathologies were observed: 15 cases of hallux valgus (46%), seven with central metatarsalgia (21%) and one with Morton's neuroma (3%). For all these cases, surgical treatments for the pathologies were performed concomitantly with the correction of the tailor's bunion.

The data used to evaluate the subjective and radiographic results studied were collected preoperatively and 6 months after surgery.

### Statistical Methodology

Initially, the data were descriptively analyzed using summary measures such as mean and standard deviation. To assess the effect of the intervention at a single evaluation timepoint on the following parameters: fifth metatarsophalangeal angle (AMF-5), fourth and fifth intermetatarsal angle (AIM4-5), the Lesser Metatarsophalangeal-Interphalangeal Scale of the American Orthopaedic Foot and Ankle Society (AOFAS), and the visual analog pain scale (VAS), we employed multilevel linear regression models. In this model, we considered feet and patients as levels 1 and 2, respectively, taking into account the potential dependence between foot observations within the same patient and the two measurements resulting from the initial and final evaluations.

The multilevel linear regression model presents normality in data distribution as one of its assumptions, which was verified using the Kolmogorov-Smirnov test.

For all statistical tests, a significance level of 5% was used. The analyzes were carried out using the statistical package SPSS 20.0 and STATA 17.

### Radiographic Measurements

Radiographs of the feet were taken in anteroposterior (AP) and lateral (L) weight-bearing views, following the standard used in our service. 10 In the study, the AMF-5 and AIM4–5 angles were analyzed, with values lower than 14° and 10°, respectively, being considered normal. 7

### Surgical technique

Patients were placed in a supine position, with the foot to be operated on slightly off the table, while the contralateral foot was supported on a support at the side.

Surgeries were performed under locoregional anesthesia (penta foot block) or spinal anesthesia. The procedures were carried out under radioscopy with the aid of a motor, rasps and cutting and grinding mills.

In total, 12 Sponsel oblique osteotomies (three associated with exostectomy) and 20 MWO osteotomies (10 associated with exostectomy) were performed.

### Sponsel Technique

**Fig. 1:**
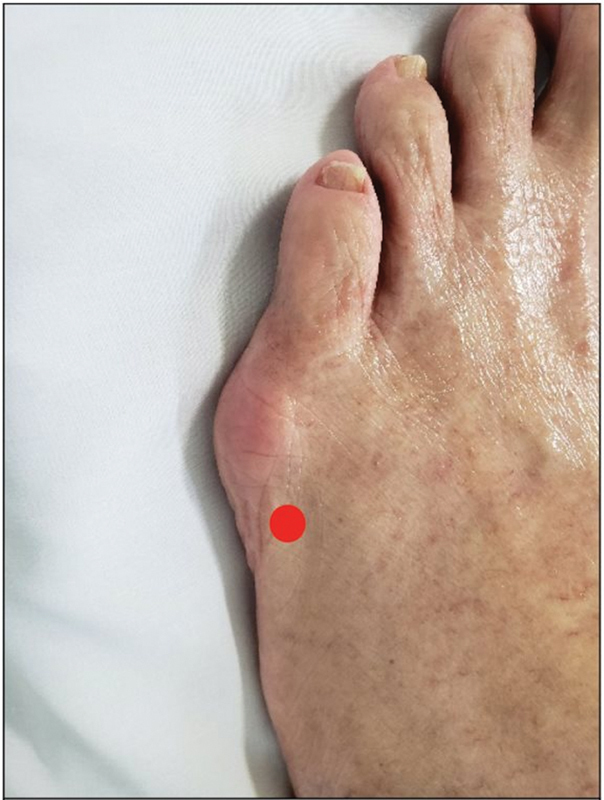
Entry point using the Sponsel technique.

The technique begins with a small incision (2mm) in the infero-lateral region proximal to the head of the 5th metatarsal bone ( Fig. 1 ), heading towards the lateral bone cortex. Next, the joint capsule is detached from the metatarsal head and, with the help of a rasp, a safe working area is created.

**Fig. 2:**
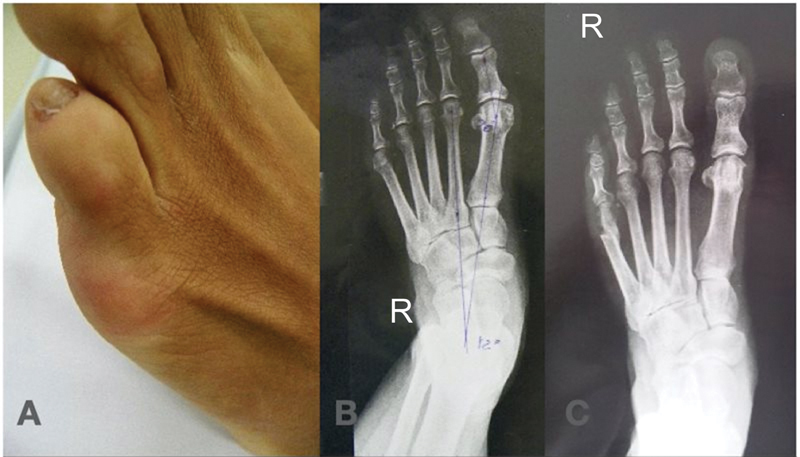
Clinical image ( **A** ). Preoperative x-ray ( **B** ). Postoperative x-ray ( **C** ).

The osteotomy occurs in the metaphyseal region, from distal to proximal, with an angle of 30° in relation to the metatarsal axis and from dorsal to plantar, which allows for proximal, dorsal and medial sliding of the head. Internal fixation is not necessary ( Fig. 2 ).

### Wedge osteotomy (MWO)

**Fig. 3:**
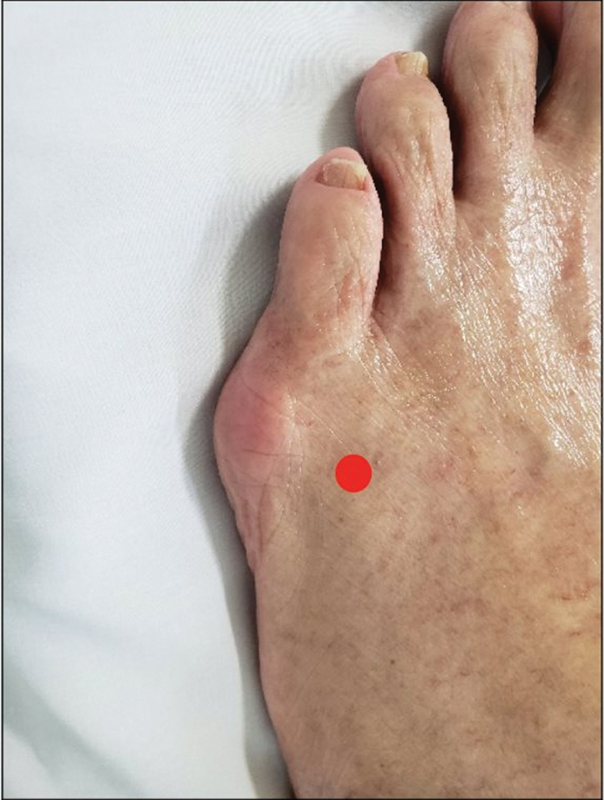
Wedge osteotomy entry point.

**Fig. 4:**
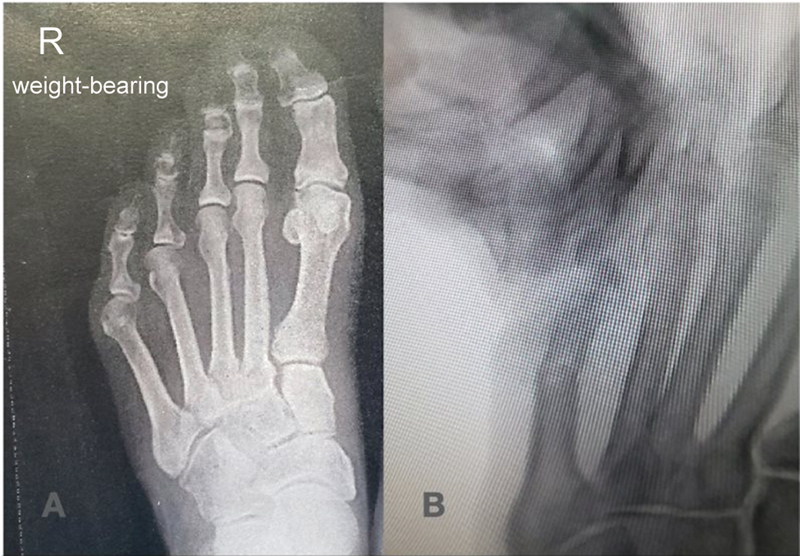
Preoperative x-ray ( **A** ). Intraoperative wedge osteotomy closure ( **B** ).

This process begins with a small incision (2 mm) in the dorsomedial region proximal to the head of the 5th metatarsal, going to the medial cortical bone ( Fig. 3 ). The osteotomy is performed under a medial to lateral orientation seeking to preserve the lateral cortex in order to create a medial closing wedge to correct the deformity ( Fig. 4 ).

After the osteotomy is completed, the percutaneous accesses are sutured and the dressing begins. Proper dressing is essential for post-surgical success, as it positions the foot in such a way as to correct the deformity, seeking medial displacement of the head of the 5th metatarsal.

### Postoperative

Patients are discharged from hospital on the same day as the surgical procedure and are instructed to walk using rigid-soled sandals. Outpatient visits are carried out every seven days to change dressings, until four weeks have passed. After this period, patients are advised to continue changing dressing at home until the eighth week, when they will be able to wear comfortable shoes.

### Subjective Results

In addition to radiographic evaluations, all patients were interviewed pre- and postoperatively using the Visual Analogue Pain Scale (VAS), 11 which consists of grading pain intensity on a scale from 0 to 10, with 0 being painless and 10 being more intense pain, and submitted to the AOFAS Lesser Metatarsophalangeal-Interphalangeal Scale questionnaire, 12 translated and adapted to Portuguese, which consists of a subjective and functional analysis, which totals 100 points, considering pain (40 points), function (45 points) and alignment (15 points). All participating patients were informed about the objectives of the work and asked to sign informed consent. This work was previously submitted and approved for analysis by the Ethics and Research Committee of the University of Taubaté (UNITAU), under opinion number 5,761,205.

## Results

Information from 29 patients was considered, whose average age was 50.7 years (SD = 14.9 years), with a minimum age of 14 years and a maximum of 84 years.

It was noted that the majority of patients (89.7%) were female. For three patients, the deformity was bilateral, thus totaling 32 feet, and average follow-up time was 36.4 months (SD= 36.9 months) ( Table 1 ).

**Table 1:** Patient characteristics and foot follow-up time by type of intervention

	Wedge osteotomy	SPONSEL	Total	*p*
**Patients**	N = 17 (58,6%)	N = 12 (41,4%)	N = 29	
**Gender, n(%)**				0,246
Masculine	3 (17,6)	0 (0,0)	3 (10,3)	
Feminine	14 (82,4)	12 (100,0)	26 (89,7)	
**Age (years)**				0,185 ^a^
Mean ± SD	47,9 ± 18,1	54,6 ± 7,6	50,7 ± 14,9	
Median (Min - Max)	45,0 (14,0 to 84,0)	56,0 (42,0 to 64,0)	51,0 (14,0 to 84,0)	
**Feet**	N = 20 (62,5%)	N = 12 (37,5%)	N = 32	
**Follow-up time (months)**				0,076 ^a^
Mean ± SD	25,2 ± 17,0	55,2 ± 52,1	36,4 ± 36,9	
Median (Min - Max)	20,0 (6,0 to 54,0)	30,5 (6,0 to 128,0)	22,0 (6,0 to 128,0)	

p - descriptive level of Fisher's Exact test and Student's t( ^a^ ).

According to Tables 2 and 3 , no interaction effect was observed for the AIM4–5 correction (p = 0.189), indicating that the mean variations were similar for both groups. Furthermore, no differences were found in the means between the intervention groups in the two assessments (p = 0.374). Likewise, there were no differences in the mean variation of the AMF-5 in relation to the type of intervention (p = 0.062). However, an average reduction in the angles evaluated in both surgical techniques was observed (p < 0.001). The mean AIM4–5 correction in the Sponsel technique was 13.4° preoperatively to 9.25° postoperatively, while in the MWO it was 12.6° to 7.15° at the final evaluation. The average reduction in AMF-5 was 10.5° using the Sponsel technique and 14.8° using the MWO.

**Table 2:** Summary measurements of intermetatarsal angles 4–5 ( ^o^ ) by assessment timepoint, according to type of intervention

	Pre	Post	Post – Pre	*p*		
	Mean ± SD	Mean ± SD	Mean ± SD	Treatment	Time	Interaction between Treatment and Time
** Intermetatarsal angles 4–5 ( ^o^ ) **				0,374	<0,001	0,189
Wedge osteotomy	12,60 ± 2,14	7,15 ± 1,50	−5,45 ± 2,98			
SPONSEL	13,42 ± 4,08	9,25 ± 2,90	−4,17 ± 2,33			

p - descriptive level of treatment effects, time and interaction between treatment and time.

**Table 3:** Summary measurements of metatarsophalangeal angle, AOFAS and Pain score by assessment timepoint, according to type of intervention

	Pre	Post	Post – Pre	*p*		
	Mean ± SD	Mean ± SD	Mean ± SD	Treatment	Time	Interaction between Treatment and Time
** Metatarsophalangeal Angle ( ^o^ ) **				0,174	<0,001	0,062
Wedge osteotomy	16,20 ± 7,27	1,40 ± 6,18	−14,80 ± 7,05			
SPONSEL	15,50 ± 8,33	5,00 ± 8,00	−10,50 ± 5,45			
**AOFAS**				<0,001	<0,001	<0,001
Wedge osteotomy	36,80 ± 8,24	93,75 ± 4,25	56,95 ± 9,58			
SPONSEL	52,83 ± 10,75	90,58 ± 7,70	37,75 ± 13,05			
**Pain**				0,002	<0,001	<0,001
Wedge osteotomy	9,0 ± 0,6	0,8 ± 0,7	−8,2 ± 0,8			
SPONSEL	8,0 ± 1,3	0,9 ± 0,7	−7,1 ± 1,2			

p - descriptive level of treatment effects, time and interaction between treatment and time.

Wedge osteotomy versus SPONSEL: AOFAS - pre: p < 0.001 and post: 0.245; Pain - pre: p = 0.002; post: 0.539.

Pre versus Post - Wedge osteotomy: AOFAS - p < 0.001 and SPONSEL: p < 0.001; Pain - p < 0.001 and SPONSEL: *p* < 0,001.

For both surgical techniques, the increase in the AOFAS score and the decrease in pain on the VAS scale stand out. However, MWO obtained statistically superior results (p < 0.001) compared to Sponsel osteotomy, both in increasing the AOFAS score and decreasing pain on the VAS scale. The mean AOFAS score increase for MWO was 55.3, while for Sponsel osteotomy it was 36.9. Furthermore, MWO showed an average pain reduction on the VAS scale of 8.2, in contrast to the Sponsel technique, which recorded an average reduction of 7.1.

**Fig. 5:**
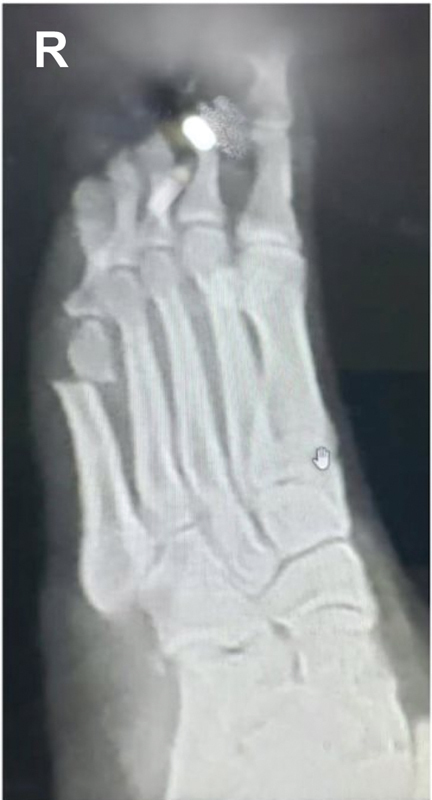
Complication of bone spike in a patient operated using the Sponsel technique.

Only two cases showed delayed consolidation, with spontaneous resolution within 6 months. We consider a delay if radiographic signs of bone consolidation are not seen within 3 months postoperatively. Additionally, a case of symptomatic bone spike formation was observed ( Fig. 5 ), which presented good resolution of symptoms after bone consolidation. In this study, no other complications were observed, such as pseudarthrosis, superficial or deep infection, recurrence, neuropraxia or hypertrophic callus.

## Discussion

Many surgical techniques have been described for the treatment of tailor's bunion over the years. 13 14 15 16 17 18 19 Recently, percutaneous techniques have gained prominence due to their less invasive approach to soft tissues, 1 2 4 20 21 with effective corrections and less postoperative pain. In this context, it is extremely important to make comparisons between different surgical techniques.

Both MWO and Sponsel osteotomy have already proven to be effective for the minimally invasive treatment of tailor's bunion, as shown by several authors. 7 22 23 24 25 By comparing two specific approaches, we seek to identify possible differences and advantages of one over the other.

When analyzing the radiographic results, we observed significant improvements in the parameters evaluated in both groups submitted to different surgical techniques. These findings are in agreement with individual studies of each technique, 7 22 23 24 25 in which similar radiographic improvements were observed.

In relation to AIM4–5, we observed an average reduction of 4.2° using the Sponsel technique, while MWO showed an average correction of 5.5°, being slightly greater. This trend was also observed in the correction of the AMF-5, in which the MWO guaranteed a greater angular correction, 14.8° versus 10.5° using the Sponsel osteotomy. We believe that this difference can be attributed to the greater capacity to medialize the head of the fifth metatarsal using the MWO technique.

However, when we directly compared the two techniques in our study, we did not find statistically significant differences in relation to the improvement in radiographic parameters. These results are consistent with the lack of comparative studies in the literature.

Regarding subjective results, both surgical techniques demonstrated a statistically significant improvement in increasing the AOFAS score and reducing pain assessed by the VAS scale, indicating the effectiveness of both approaches in relieving pain and functional improvement of the foot affected by tailor's bunion. Results similar to those obtained in other studies are available in the literature. 3 22 24 25

When comparing the techniques, we observed a significant difference in the improvement of the AOFAS score in favor of MWO in relation to Sponsel osteotomy, with an average improvement of 55.3 points versus 36.9, respectively. Furthermore, the analysis of pain reduction using the VAS scale followed the same trend, with the MWO technique presenting statistically significant better results (average reduction of 8.2 points in the OCM group versus 7.1 points in the oblique osteotomy group; p < 0.001). These results provide additional evidence that MWO may be a more effective surgical approach for treating tailor's bunion, resulting in improved function and reduced postoperative pain.

Both techniques used presented satisfactory results with few complications. Using the Sponsel technique, two cases of delayed consolidation were observed, which progressed well after the formation of bone callus around the sixth month, and one case of symptomatic bone spike formation, which also progressed with resolution of symptoms without the need for new surgical intervention ( Fig. 5 ). Using the wedge osteotomy technique, no complications were observed. Such findings, when compared with the literature, confirm the low complication rates of percutaneous techniques, especially for distal osteotomies. 3 21 23 25

Some limitations of our study should be mentioned. Both preoperative and postoperative assessments were carried out by the same team, which could generate performance bias in data analysis. The number of cases operated using both techniques could be greater, allowing a more statistically significant comparison of results.

Another limitation is that the AOFAS Lesser Metatarsophalangeal-Interphalangeal Scale questionnaire was translated and adapted into Portuguese by the researchers, as at the time of this study we have not found work with a validated translation for the lesser toes scale. Therefore, it is important to consider that the results obtained may not have been completely reliable due to the lack of validation of the translated scale. Furthermore, although the questionnaire for smaller toes was used, which has a more specific approach to tailor's bunion, it is important to highlight that other pathologies were associated and may have influenced the subjective results obtained. For a future study, we suggest including only those with tailor's bunion as a single deformity, thus analyzing the results can be more objective.

Our study was considered pertinent, presenting the comparison of the results of two percutaneous surgical techniques for the correction of tailor's bunion, both with low rates of complications and promoting a good improvement in the deformity in the operated feet.

## Conclusions

Both the wedge osteotomy (MWO) technique and Sponsel's oblique osteotomy proved to be effective in correcting and improving radiographic and clinical parameters in the treatment of tailor's bunion. However, MWO showed statistically superior results in the AOFAS scoring criteria and pain scale (VAS). Based on these findings, we started to adopt MWO as the preferred technique for the surgical approach to tailor's bunion in our service. However, it is important to highlight that each case must be evaluated individually, taking into account the patient's characteristics, the degree of deformity and the surgeon's experience, in order to define the most appropriate technique to be used.
