# Supplementary material for: Percutaneous Surgery for Tailor's Bunion: A Comparative Study
Source: Rev Bras Ortop (Sao Paulo). 2023 Dec 5;59(5):e730–6. doi: 10.1055/s-0043-1776292 (PMC11624943; doi:10.1055/s-0043-1776292)
Supplement: Related texts

# Research article: Tratamento cirúrgico do joanete do alfaiate por técnica percutânea: Um estudo comparativo

**Keywords:** joanete do alfaiate/diagnóstico por imagem, joanete do alfaiate/cirurgia, ossos do metatarso, resultado do tratamento

## Abstract

**Objetivos** Analisar e comparar os resultados clínicos e radiográficos da correção do joanete do alfaiate, utilizando-se duas técnicas cirúrgicas percutâneas, técnica de osteotomia oblíqua de Sponsel e osteotomia em cunha medial da metáfise distal. Os resultados foram avaliados individualmente e comparativamente através do *Lesser Metatarsophalangeal-Interphalangeal Scale* da *American Orthopaedic Foot and Ankle Society* (AOFAS), Escala Visual Analógica de Dor (VAS) e radiograficamente, analisando-se os ângulos: ângulo intermetatársico IV-V (AIM4–5) e ângulo metatarsofalangico do 5° raio (AMF-5).

**Métodos** Trata-se de um estudo retrospectivo no período de maio de 2011 a fevereiro de 2022. Foram operados 32 pés, destes, em 12 pés foram utilizadas a técnica de Sponsel e em 20 pés a osteotomia em cunha medial da metáfise distal do 5° metatársico.

**Resultados** Ambas as técnicas cirúrgicas empregadas mostraram melhora significativa na correção dos ângulos AIM4–5 e AMF-5 ( *p* < 0,001), todavia, não observamos significância estatística comparando-se as técnicas. Já em relação ao AOFAS e VAS, ambas as técnicas apresentaram resultados satisfatórios. Porém, a osteotomia em cunha apresentou resultados significativamente melhores quando comparados a técnica de *Sponsel* ( *p* < 0,001).

**Conclusões** Ambas as técnicas percutâneas utilizadas para correção do joanete do alfaiate conferiram melhora significativa das angulações radiográficas e dos scores avaliados, com baixo índice de complicações, mostrando-se boas opções para tratamento desta patologia. Quando comparadas, a osteotomia em cunha medial parece conferir melhores resultados dos parâmetros clínicos analisados.

## Introdução

O joanete do alfaiate caracteriza-se por uma deformidade na região da cabeça do quinto metatarso com proeminência dolorosa lateral, dorsolateral ou plantar. A sua etiologia é multifatorial, 1 estando associada tanto a fatores anatômicos como estruturais. 2 3 4

O tratamento inicial consiste na aplicação de medidas conservadoras, 5 enquanto o tratamento cirúrgico é indicado quando ocorre falha no tratamento conservador. Diversas técnicas cirúrgicas foram descritas para o tratamento desta deformidade, que consistem basicamente em exostectomias, ressecções ósseas e osteotomias metatársicas (distais, diafisárias e proximais). Essas técnicas são indicadas após uma avaliação clínica e radiográfica, baseada na classificação do tipo de deformidade. 1 2 4 6 7

Inicialmente descrita por Isham 8 e De Prado et al., 9 a cirurgia percutânea vem ganhando espaço e popularidade nos últimos anos, mostrando-se um procedimento útil para o tratamento do joanete do alfaiate de forma minimamente invasiva. 7

O objetivo do presente estudo é comparar os aspectos clínicos e radiográficos de pacientes submetidos a tratamento cirúrgico do joanete do alfaiate por duas técnicas percutâneas diferentes: a osteotomia oblíqua de Sponsel 1 e a osteotomia em cunha medial da metáfise distal do 5° osso metatársico (OCM). 5

## Métodos

Durante o período compreendido entre maio de 2011 a fevereiro de 2022, foram operados 29 pacientes portadores de Joanete do Alfaiate, sendo 3 bilaterais, totalizando assim 32 pés. Os procedimentos foram realizados em um Hospital Municipal Universitário e em uma clínica privada de um dos autores.

Neste estudo foram incluídos os casos sintomáticos refratários ao tratamento conservador, com tempo mínimo de seguimento de 6 meses de pós-operatório. Além disso, foram excluídos os pacientes com as seguintes características: histórico de cirurgia prévia no 5° raio, portadores de doenças vasculares e/ou reumatoides e portadores de calosidades do 5° osso metatársico que não geravam alterações angulares radiográficas, sendo estes, submetidos apenas à exostectomia.

Neste contexto, entre os 32 pés portadores de joanete do alfaiate avaliados no estudo, foram observadas outras patologias associadas: 15 casos de hálux valgo (46%), sete com metatarsalgia central (21%) e um de neuroma de Morton (3%). Para todos esses casos, os tratamentos cirúrgicos das patologias foram realizados concomitantemente com a correção do joanete do alfaiate.

Os dados utilizados para avaliação dos resultados subjetivos e radiográficos estudados foram coletados no pré-operatório e 6 meses após a cirurgia.

## Metodologia Estatística

Inicialmente, os dados foram analisados descritivamente por meio de medidas de resumo, como média e desvio padrão. Para avaliar o efeito da intervenção em um único momento de avaliação nos seguintes parâmetros: ângulo metatarsofalângico quinto raio (AMF-5), ângulo intermetatarsal 4° e 5° (AIM4–5), *Lesser Metatarsophalangeal-Interphalangeal Scale* da *American Orthopaedic Foot and Ankle Society* (AOFAS) e escala analógica de dor (VAS), utilizamos modelos de regressão linear multinível. Nesse modelo, consideramos os pés e os pacientes como nível 1 e 2, respectivamente, levando em conta a possível dependência entre as observações dos pés dentro de um mesmo paciente e as duas medidas resultantes das avaliações inicial e final.

O modelo de regressão linear multinível apresenta como um dos pressupostos, a normalidade na distribuição dos dados, o qual foi verificado utilizando-se o teste de Kolmogorov-Smirnov.

Para todos os testes estatísticos foi utilizado um nível de significância de 5%. As análises foram realizadas utilizando-se o pacote estatístico SPSS 20.0 e STATA 17.

## Medidas Radiográficas

As radiografias dos pés foram realizadas nas incidências anteroposterior (AP) e em perfil (P) com carga, seguindo o padrão utilizado em nosso serviço. 10 No estudo, foram analisados os ângulos AMF-5 e AIM4–5, sendo considerados normais, os valores inferiores a 14° e 10°, respectivamente. 7

## Técnica Cirúrgica

Os pacientes foram colocados em posição supina, com o pé a ser operado levemente fora da mesa, enquanto o pé contralateral apoiava-se em um suporte ao lado.

As cirurgias foram realizadas sob anestesia loco-regional (penta bloqueio do pé) ou raquianestesia. Os procedimentos foram realizados sob radioscopia com auxílio de motor, raspas e fresas de corte e desbastamento.

Ao todo foram realizadas 12 osteotomias oblíquas de Sponsel (três associadas à exostectomia) e 20 de OCM (10 associadas à exostectomia).

## Técnica de Sponsel

A técnica inicia com uma pequena incisão (2mm) na região ínfero-lateral proximal à cabeça do 5° osso metatársico ( Fig. 1 ), direcionando-se até a cortical óssea lateral. Na sequência, realiza-se o descolamento da cápsula articular da cabeça metatársica e com auxílio de uma raspa cria-se uma zona de trabalho segura.

**Fig. 1:** Ponto de entrada pela técnica de Sponsel.

A osteotomia ocorre na região metafisária, de distal para proximal, com angulação de 30° em relação ao eixo do metatársico e de dorsal para plantar, que possibilita um deslizamento proximal, dorsal e medial da cabeça. A fixação interna não se faz necessária ( Fig. 2 ).

**Fig. 2:** Imagem clínica ( **A** ). Raio-x pré-operatório ( **B** ). Raio-x pós-operatório ( **C** ).

## Osteotomia em Cunha (OCM)

Esse processo inicia-se com uma pequena incisão (2mm) na região dorsomedial proximal à cabeça do 5° metatarso, dirigindo-se até a cortical óssea medial ( Fig. 3 ). A osteotomia é realizada sob orientação medial para lateral buscando preservar a cortical lateral, a fim de criar uma cunha de fechamento medial para correção da deformidade ( Fig. 4 ).

**Fig. 3:** Ponto de entrada osteotomia em cunha.

**Fig. 4:** Raio-x pré-operatório ( **A** ). Fechamento da osteotomia em cunha intraoperatório ( **B** ).

Após a conclusão da osteotomia, é realizada a sutura dos acessos percutâneos e inicia-se o curativo. A confecção adequada do curativo é fundamental para o sucesso pós-cirúrgico, pois posiciona pé de forma a corrigir a deformidade, buscando o deslocamento medial da cabeça do 5° metatársico.

## Pós-operatório

Os pacientes recebem alta hospitalar no mesmo dia do procedimento cirúrgico e são orientados a deambular com o uso de sandália de solado rígido. Os retornos ambulatoriais são realizados a cada sete dias para a troca dos curativos, até se completarem quatro semanas. Após esse período, os pacientes são orientados a manter as trocas de curativos em domicílio até a oitava semana, quando então, poderão utilizar calçados confortáveis.

## Resultados Subjetivos

Além das avaliações radiográficas, todos os pacientes foram entrevistados no pré e pós-operatórios através da Escala Visual Analógica de Dor (VAS), 11 que consiste na graduação da intensidade da dor, em escala de 0 a 10, sendo 0 para indolor e 10 uma dor mais intensa, e submetidos ao questionário *Lesser Metatarsophalangeal-Interphalangeal Scale* da AOFAS, 12 traduzido e adaptado ao português, que consiste em uma análise subjetiva e funcional, da qual totaliza 100 pontos, considerando dor (40 pontos), função (45 pontos) e alinhamento (15 pontos). Todos os pacientes participantes foram esclarecidos sobre os objetivos do trabalho e solicitado a assinatura do consentimento livre e esclarecido. Este trabalho foi previamente submetido e aprovado à análise pelo Comitê de Ética e Pesquisa da Universidade de Taubaté (UNITAU), sob parecer de número 5.761.205.

## Resultados

Foram consideradas as informações de 29 pacientes, cuja média de idade foi 50,7 anos (DP = 14,9 anos), com uma idade mínima de 14 anos e máxima de 84 anos.

Notou-se que a maioria dos pacientes (89,7%), correspondiam ao sexo feminino. Para três pacientes a deformidade era bilateral, totalizando-se assim, 32 pés, cujo tempo médio de seguimento foi de 36,4 meses (DP= 36,9 meses) ( Tabela 1 ).

**Tabela 1:** Características dos pacientes e tempo de seguimento dos pés por tipo de intervenção

	Osteotomia em cunha	SPONSEL	Total	*p*
**Pacientes**	N = 17 (58,6%)	N = 12 (41,4%)	N = 29	
**Gênero, n(%)**				0,246
Masculino	3 (17,6)	0 (0,0)	3 (10,3)	
Feminino	14 (82,4)	12 (100,0)	26 (89,7)	
**Idade (anos)**				0,185 ^a^
Média ± DP	47,9 ± 18,1	54,6 ± 7,6	50,7 ± 14,9	
Mediana (Mín - Max)	45,0 (14,0 a 84,0)	56,0 (42,0 a 64,0)	51,0 (14,0 a 84,0)	
**Pés**	N = 20 (62,5%)	N = 12 (37,5%)	N = 32	
**Tempo de seguimento (meses)**				0,076 ^a^
Média ± DP	25,2 ± 17,0	55,2 ± 52,1	36,4 ± 36,9	
Mediana (Mín - Max)	20,0 (6,0 a 54,0)	30,5 (6,0 a 128,0)	22,0 (6,0 a 128,0)	

p - nível descritivo do teste Exato de Fisher e t de Student( ^a^ ).

De acordo com as Tabelas 2 e 3 , não foi observado efeito de interação para a correção do AIM4–5 ( *p* = 0,189), indicando que as variações médias foram similares para ambos os grupos. Além disso, não foram encontradas diferenças nas médias entre os grupos de intervenção nas duas avaliações ( *p* = 0,374). Da mesma forma, não houve diferenças nas médias de variação do AMF-5 em relação ao tipo de intervenção ( *p* = 0,062). No entanto, observou-se uma redução média nos ângulos avaliados em ambas as técnicas cirúrgicas ( *p* < 0,001). A correção do AIM4–5 médio na técnica de Sponsel foi de 13,4° no pré-operatório para 9,25° no pós-operatório, enquanto na OCM foi de 12,6° para 7,15° na avaliação final. Já a redução média do AMF-5 foi de 10,5° pela técnica de Sponsel e de 14,8° pela OCM.

**Tabela 2:** Medidas-resumo dos ângulos intermetatarsais 4–5 ( ^o^ ) por momento de avaliação, segundo tipo de intervenção

	Pré	Pós	Pós – Pré	*p*		
	Média ± DP	Média ± DP	Média ± DP	Tratamento	Tempo	Interação entre Tratamento e Tempo
** Ângulos intermetatarsais 4–5 ( ^o^ ) **				0,374	<0,001	0,189
Osteotomia em cunha	12,60 ± 2,14	7,15 ± 1,50	−5,45 ± 2,98			
SPONSEL	13,42 ± 4,08	9,25 ± 2,90	−4,17 ± 2,33			

p - nível descritivo dos efeitos de tratamento, tempo e interação entre tratamento e tempo.

**Tabela 3:** Medidas-resumo de ângulo metatarsofalangico, AOFAS e escore de Dor por momento de avaliação, segundo tipo de intervenção

	Pré	Pós	Pós – Pré	*p*		
	Média ± DP	Média ± DP	Média ± DP	Tratamento	Tempo	Interação entre Tratamento e Tempo
** Ângulo metatarsofalangico ( ^o^ ) **				0,174	<0,001	0,062
Osteotomia em cunha	16,20 ± 7,27	1,40 ± 6,18	−14,80 ± 7,05			
SPONSEL	15,50 ± 8,33	5,00 ± 8,00	−10,50 ± 5,45			
**AOFAS**				<0,001	<0,001	<0,001
Osteotomia em cunha	36,80 ± 8,24	93,75 ± 4,25	56,95 ± 9,58			
SPONSEL	52,83 ± 10,75	90,58 ± 7,70	37,75 ± 13,05			
**Dor**				0,002	<0,001	<0,001
Osteotomia em cunha	9,0 ± 0,6	0,8 ± 0,7	−8,2 ± 0,8			
SPONSEL	8,0 ± 1,3	0,9 ± 0,7	−7,1 ± 1,2			

p - nível descritivo dos efeitos de tratamento, tempo e interação entre tratamento e tempo.

Osteotomia em cunha versus SPONSEL: AOFAS - pré: *p* < 0,001 e pós: 0,245; Dor - pré: *p* = 0,002; pós: 0,539.

Pré versus Pós - Osteotomia em cunha: AOFAS - *p* < 0,001 e SPONSEL: *p* < 0,001; Dor - *p* < 0,001 e SPONSEL: *p* < 0,001.

Para ambas as técnicas cirúrgicas, destaca-se o aumento da pontuação AOFAS e diminuição de dor na escala VAS. No entanto, a OCM obteve resultados estatisticamente superiores ( *p* < 0,001) em comparação com a osteotomia de Sponsel, tanto no aumento da pontuação AOFAS quanto na diminuição da dor na escala VAS. O aumento médio da pontuação AOFAS para a OCM foi de 55,3, enquanto para a osteotomia de Sponsel foi de 36,9. Além disso, a OCM apresentou uma redução média da dor na escala VAS de 8,2, em contraste com a técnica de Sponsel, que registrou uma redução média de 7,1.

Apenas dois casos apresentaram retardo de consolidação, com resolução espontânea em 6 meses. Consideramos retardo a não visualização de sinais radiográficos de consolidação óssea em até 3 meses de pós-operatório. Adicionalmente, foi observado um caso de formação de espícula óssea sintomática ( Fig. 5 ), que apresentou boa resolução dos sintomas após a consolidação óssea. Neste estudo não foram observadas outras complicações como pseudartrose, infecção superficial ou profunda, recidiva, neuropraxias ou calo hipertrófico.

**Fig. 5:** Complicação de espícula óssea em paciente operado pela técnica de Sponsel.

## Discussão

Muitas técnicas cirúrgicas têm sido descritas para o tratamento do joanete do alfaiate ao longo dos anos. 13 14 15 16 17 18 19 Recentemente, as técnicas percutâneas têm ganhado destaque devido à sua abordagem menos invasiva às partes moles, 1 2 4 20 21 com correções efetivas e menor dor no pós-operatório. Nesse contexto, é de suma importância realizar comparações entre diferentes técnicas cirúrgicas.

Tanto a OCM, quanto a osteotomia de Sponsel, já se mostraram efetivas para o tratamento minimamente invasivo do joanete do alfaiate, conforme mostrado por diversos autores. 7 22 23 24 25 Ao comparar duas abordagens específicas, buscamos identificar possíveis diferenças e vantagens de uma sobre a outra.

Ao analisar os resultados radiográficos, observamos melhorias significativas nos parâmetros avaliados em ambos os grupos submetidos às diferentes técnicas cirúrgicas. Esses achados estão em concordância com estudos individuais de cada técnica, 7 22 23 24 25 nos quais foram observadas melhorias radiográficas semelhantes.

Em relação ao AIM4–5, observamos uma redução média de 4,2° pela técnica de Sponsel, enquanto a OCM apresentou uma correção média de 5,5°, sendo ligeiramente maior. Tal tendência também foi observada na correção do AMF-5, em que a OCM garantiu uma maior correção angular, 14,8° contra 10,5° pela osteotomia de Sponsel. Acreditamos que essa diferença pode ser atribuída à maior capacidade de medialização da cabeça do quinto metatarso pela técnica de OCM.

No entanto, quando comparamos diretamente as duas técnicas em nosso estudo, não encontramos diferenças estatisticamente significantes em relação à melhora dos parâmetros radiográficos. Esses resultados são consistentes com a falta de estudos comparativos na literatura.

Quanto aos resultados subjetivos, ambas as técnicas cirúrgicas demonstraram uma melhora estatisticamente significativa no aumento da pontuação AOFAS e na redução da dor avaliada pela escala VAS, indicando eficácia de ambas as abordagens no alívio da dor e na melhora funcional do pé afetado pelo joanete do alfaiate. Resultados semelhantes aos obtidos em outros estudos disponíveis na literatura. 3 22 24 25

Ao compararmos as técnicas, observamos uma diferença significativa na melhora da pontuação AOFAS em favor da OCM em relação a osteotomia de Sponsel, com uma melhora média de 55,3 pontos contra 36,9, respectivamente. Além disso, a análise da redução da dor pela escala VAS seguiu a mesma tendência, com a técnica de OCM apresentando melhores resultados estatisticamente significativos (redução média de 8,2 pontos no grupo da OCM versus 7,1 pontos no grupo da osteotomia oblíqua; *p* < 0,001). Esses resultados fornecem evidências adicionais que a OCM pode ser uma abordagem cirúrgica mais efetiva para o tratamento do joanete do alfaiate, resultando em uma melhor função e redução da dor no pós-operatório.

Ambas as técnicas empregadas apresentaram resultados satisfatórios com poucas complicações. Pela técnica de Sponsel foram observados dois casos de retardo de consolidação, que evoluíram bem após a formação de calo ósseo por volta do sexto mês, e um caso de formação de espícula óssea sintomática, que também evoluiu com resolução dos sintomas sem a necessidade de nova intervenção cirúrgica ( Fig. 5 ). Pela técnica de osteotomia em cunha não foram observadas complicações. Tais achados, quando confrontados com a literatura, confirmam os baixos índices de complicações das técnicas percutâneas, sobretudo para osteotomias distais. 3 21 23 25

Algumas limitações do nosso estudo devem ser mencionadas. Tanto as avaliações pré-operatórias quanto as pós-operatórias foram realizadas pela mesma equipe, o que pode gerar um viés de desempenho na análise dos dados. O número de casos operados, por ambas as técnicas, poderia ser maior, permitindo uma comparação dos resultados de forma mais significativa estatisticamente.

Outra limitação é que o questionário *Lesser Metatarsophalangeal-Interphalangeal Scale* da AOFAS foi traduzido e adaptado para língua portuguesa pelos pesquisadores, pois até o momento deste estudo não encontramos trabalho com uma tradução validada para a escala dos dedos menores. Portanto, é importante considerar que os resultados obtidos podem não ter sido totalmente confiáveis devido à falta de validação da escala traduzida. Além disso, embora tenha sido utilizado o questionário para os dedos menores, que possui uma abordagem mais específica para joanete do alfaiate, é importante ressaltar que outras patologias estavam associadas, podendo ter influenciado nos resultados subjetivos obtidos. Para um próximo estudo sugerimos a inclusão apenas de portadores do joanete do alfaiate como deformidade única, deste modo, a análise dos resultados poderá ser mais objetiva.

Nosso estudo foi considerado pertinente, apresentando a comparação dos resultados de duas técnicas cirúrgicas percutâneas para a correção do joanete do alfaiate, ambas com baixos índices de complicações e promovendo uma boa melhora da deformidade nos pés operados.

## Conclusões

Tanto a técnica de osteotomia em cunha (OCM) quanto a osteotomia oblíqua de Sponsel se mostraram efetivas na correção e melhora dos parâmetros radiográficos e clínicos no tratamento do joanete do alfaiate. No entanto, a OCM apresentou resultados estatisticamente superiores nos critérios de pontuação AOFAS e escala de dor (VAS). Com base nesses achados, passamos a adotar a OCM como técnica preferencial para abordagem cirúrgica do joanete do alfaiate em nosso serviço. No entanto, é importante ressaltar que cada caso deve ser avaliado individualmente, levando em consideração as características do paciente, o grau da deformidade e a experiência do cirurgião, a fim de definir a técnica mais apropriada a ser empregada.
